# Effective Second-Stage Surgery for Intraorbital Abscess Caused by Inverted Papilloma: A Case Report

**DOI:** 10.7759/cureus.81148

**Published:** 2025-03-25

**Authors:** Risa Tagaya, Ryo Maruyama, Masanori Yatomi, Haruka Nishimura, Kiyoaki Tsukahara

**Affiliations:** 1 Otolaryngology-Head and Neck Surgery, Tokyo Medical University, Tokyo, JPN; 2 Otolaryngology-Head and Neck Surgery, Tokyo medical University, Tokyo, JPN

**Keywords:** abscess, frontal sinus, inverted papilloma, paranasal sinus neoplasms, vision disorders

## Abstract

An intraorbital abscess is an urgent condition that can cause visual impairment and intracranial complications. Here, we reported an intraorbital abscess caused by a frontal sinus inverted papilloma. If an infection from a nasal or paranasal sinus tumor is suspected, treatment strategies vary depending on whether the tumor is benign or malignant. Additionally, if emergency drainage is required, an extra nasal or an intranasal approach or a combination of both is selected depending on the localization and extent of the abscess. In the present case, we first performed emergency surgery using a combination of extra nasal and intranasal approaches to improve visual function, drainage, and diagnosis. After the definitive diagnosis, we were able to control both diseases through a two-stage treatment, including surgery for radical treatment. Given the effectiveness of the two-stage treatment, it could be considered for managing intraorbital abscesses.

## Introduction

Nasal intraorbital complications, such as orbital cellulitis and intraorbital abscesses, are caused by the spread of inflammation, such as sinusitis, through the gap and hematogenous route in the venous system, as the orbit is in contact with the paranasal sinuses through a paper-like plate [1-4]. Nasal intraorbital abscess due to acute sinusitis or cyst infection has been occasionally reported [4,5]. Some causes of sinusitis are secondary, and diagnosis and treatment of the causative disease are necessary [6]. Secondary causes include tumors such as inversion papillomas, and the surgical method must be selected depending on the localization and extent. An intraorbital abscess is an urgent condition that can cause visual impairment and intracranial complications and requires immediate attention. Here, we report the disease progression and treatment process of the case of an intraorbital abscess caused by a frontal inverted papilloma in which both diseases were controlled using a two-stage treatment. This case report was prepared in accordance with the CARE guidelines. We obtained the patient’s consent to publish this case report.

## Case presentation

The patient was a 31-year-old man with chief complaints of left eye pain and left upper eyelid swelling. The patient presented with pain in the left eye, swelling of the left upper eyelid, and difficulty opening the left eye. He visited the ophthalmology department of our hospital two days after symptom onset. Orbital cellulitis was suspected; the patient was admitted to the hospital on the same day, and antibiotic treatment was initiated. The selected drug was cefazolin. Three days after onset, the patient's light valve and light reflex disappeared after the appearance of diplopia, and computed tomography (CT) of his head at admission revealed soft tissue shadows in the sinuses that were suspected to be an abscess, and he was referred to our department.

The patient’s past medical history included hyperuricemia, and he had no particular allergies. His vital signs were clear, he was conscious, and his body temperature was 38.0ºC, blood pressure was 162/104 mmHg, pulse rate was 105 beats/min, and SpO2 was 94% on room air. Blood tests revealed a white blood cell count of 17.5 x 10^3^/μL and a C-reactive protein of 4.5 mg/dL with an elevated inflammatory response. There were no other noteworthy items.

**Figure 1:**
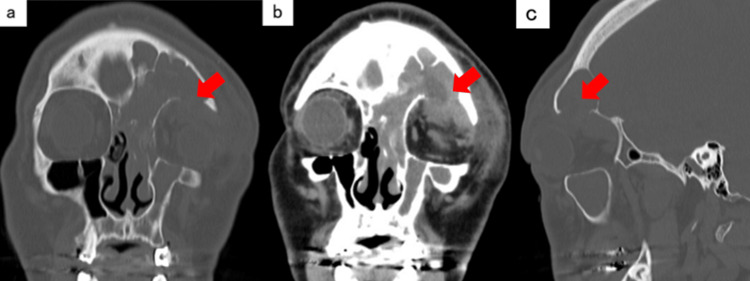
Orbital plain CT a) Coronal bone condition. b) Coronal soft tissue condition. c) Sagittal bone condition. Soft shadows filling the left ethmoid, frontal, and maxillary sinuses, bone destruction above the orbit, and a low-density area with irregular margins in the upper part of the orbit are observed. The arrows indicate the destruction of the bone above the orbit and the low-density area with irregular edges in the upper part of the orbit.

**Figure 2:**
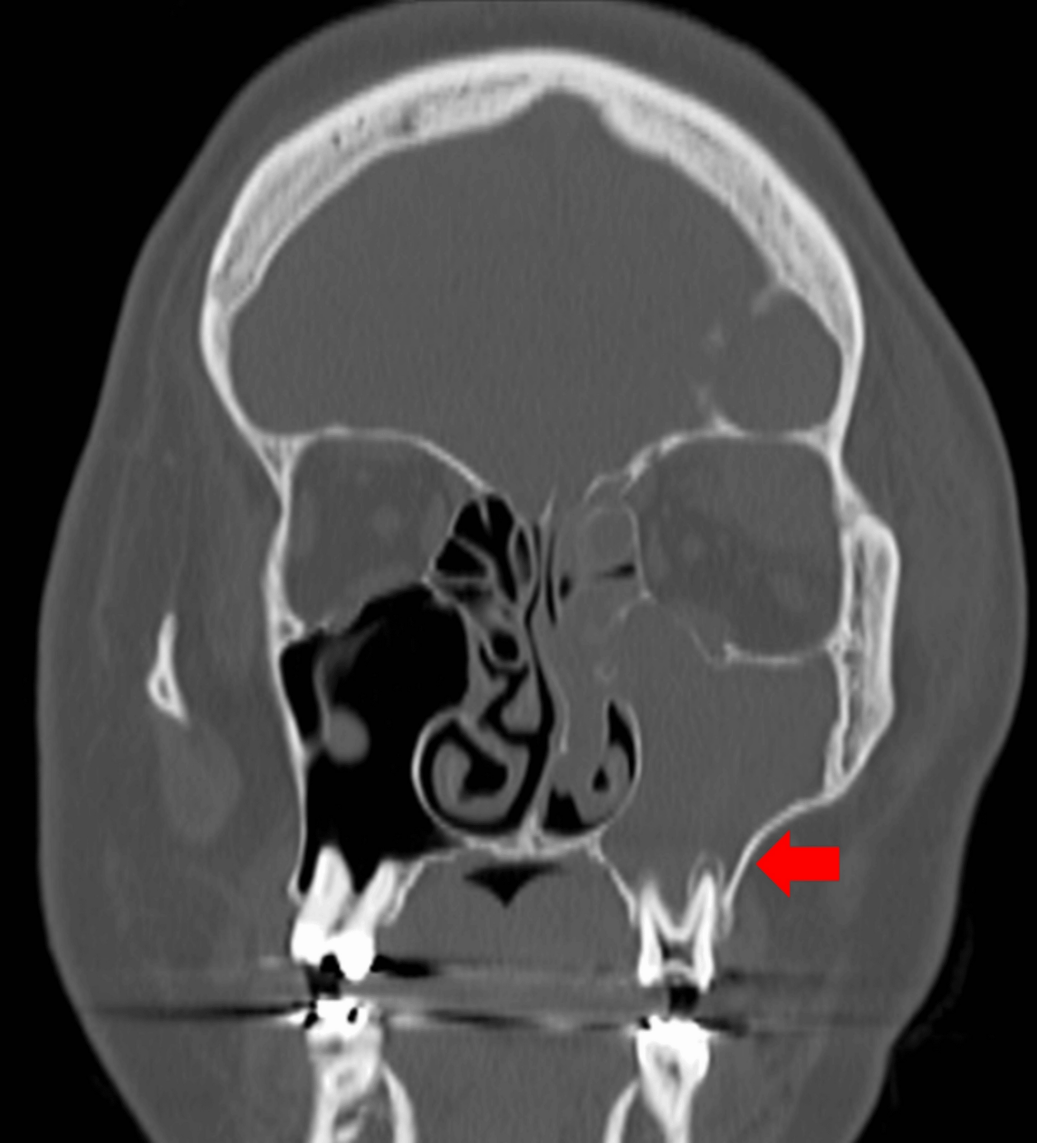
Sinus simple CT coronary section Bone resorption around the root apex of the left maxillary sinus and bone loss on the lower wall of the maxillary sinus are observed. The arrow indicates a decrease in bone resorption in the left maxillary molar root area.

Orbital plain computed tomography (CT) (Figures 1a-1c, 2) revealed soft tissue shadows filling the left ethmoid, frontal, and maxillary sinuses, bone destruction on the upper wall of the orbit, and irregular margins in the upper part of the orbit. A low-absorption region was observed. Bone resorption around the root apex of the left maxillary sinus and the loss of the lower wall of the maxillary sinus were observed.

**Figure 3:**
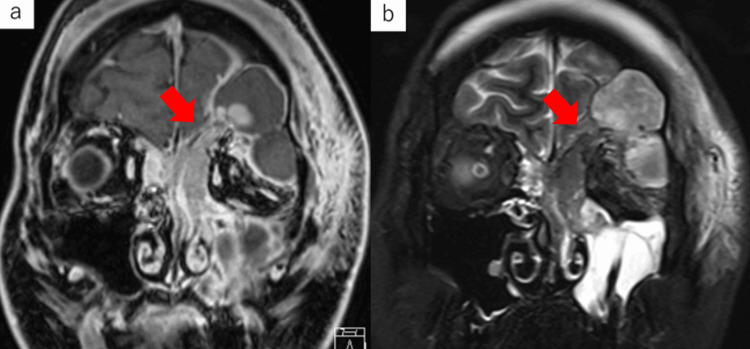
Contrast-enhanced MRI coronal section of the head a) T1-weighted image. b) T2-weighted image. An irregularly shaped mass lesion is observed extending from the left frontal sinus to the ethmoid sinus. There is a convergence site of a gyri-like structure from the nasofrontal canal to the posterior wall of the frontal sinus, which is assumed to be the base. Abscess formation is observed in the orbit, intracranial epidural area, and left maxillary sinus with low signal at T1 and a mix of low and high signals at T2. The arrow indicates the base of the nasal sinus mass.

Contrast-enhanced magnetic resonance imaging (MRI) of the head (Figures 3a, 3b) revealed an irregularly shaped mass lesion extending from the left frontal sinus to the ethmoid sinus and a convergence area of gyri-like structures from the nasofrontal canal to the posterior wall of the frontal sinus was considered basal. Abscess formation was observed in the orbit, intracranial epidural area, and left maxillary sinus with low signal at T1 and high signal at T2.

Head CT and MRI revealed a left ethmoid sinus-to-frontal sinus tumor/abscess, intraorbital abscess, and intracranial epidural abscess. The image revealed caries and the possibility that the left dentary maxillary sinus was a contributing factor to the inflammation could not be dismissed. On the same day, endoscopic sinus surgery (ESS) to open the ethmoid sinus, frontal sinus, and maxillary sinus; intraorbital abscess incision and drainage; and intranasal tumor biopsy were performed to drain the intraorbital abscess, preserve visual function, and diagnose the tumor.

The maxillary sinuses drain through the nasal cavity. At the time of emergency surgery, we prioritized visual function improvement, limited the biopsy of the tumor filling the ethmoid sinus, and decided to perform a two-stage surgery. Owing to tumor filling, it was difficult to approach the orbit from inside the nose, and because the intraorbital abscess was located superolaterally, an external incision was used for drainage.

After drainage and antibiotic therapy with ampicillin/sulbactam, the inflammatory response, eyelid swelling, and pain awareness tended to improve. Regarding visual acuity, only a light sensation appeared, and a light reflex was observed. The possibility of left maxillary sinusitis on CT could not be dismissed, and the tooth was extracted later by a dentist and oral surgeon.

**Figure 4:**
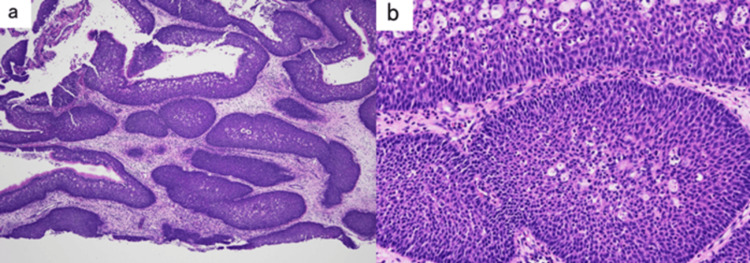
Pathological findings at the time of emergency surgery Atypical epithelial cells proliferated in an inverted manner with intussusception, and in some cases, exophytic papillary structures are observed on the surface layer. a) Hematoxylin and eosin (HE) staining at 4× magnification, b) HE staining at 20× magnification.

Pathological findings during emergency surgery showed that the dysmorphic epithelial cells proliferated mainly in an inverted manner with intussusception, and exophytic papillary structures were observed in some areas of the surface (Figures 4a, 4b). The patient was diagnosed with sinonasal inverted papilloma.

Magnetic resonance imaging (MRI) revealed that the base extended from the nasofrontal canal to the posterior wall of the frontal sinus. To remove the inverted papilloma, we decided to perform ESS to open the ethmoid sinus and frontal sinus + endoscopic enlarged frontal sinus surgery (Draf type IIb [7]) and extranasal frontal sinus surgery (Killian method [8]) 12 days after the emergency surgery, which was then performed on the patient’s eye.

**Figure 5:**
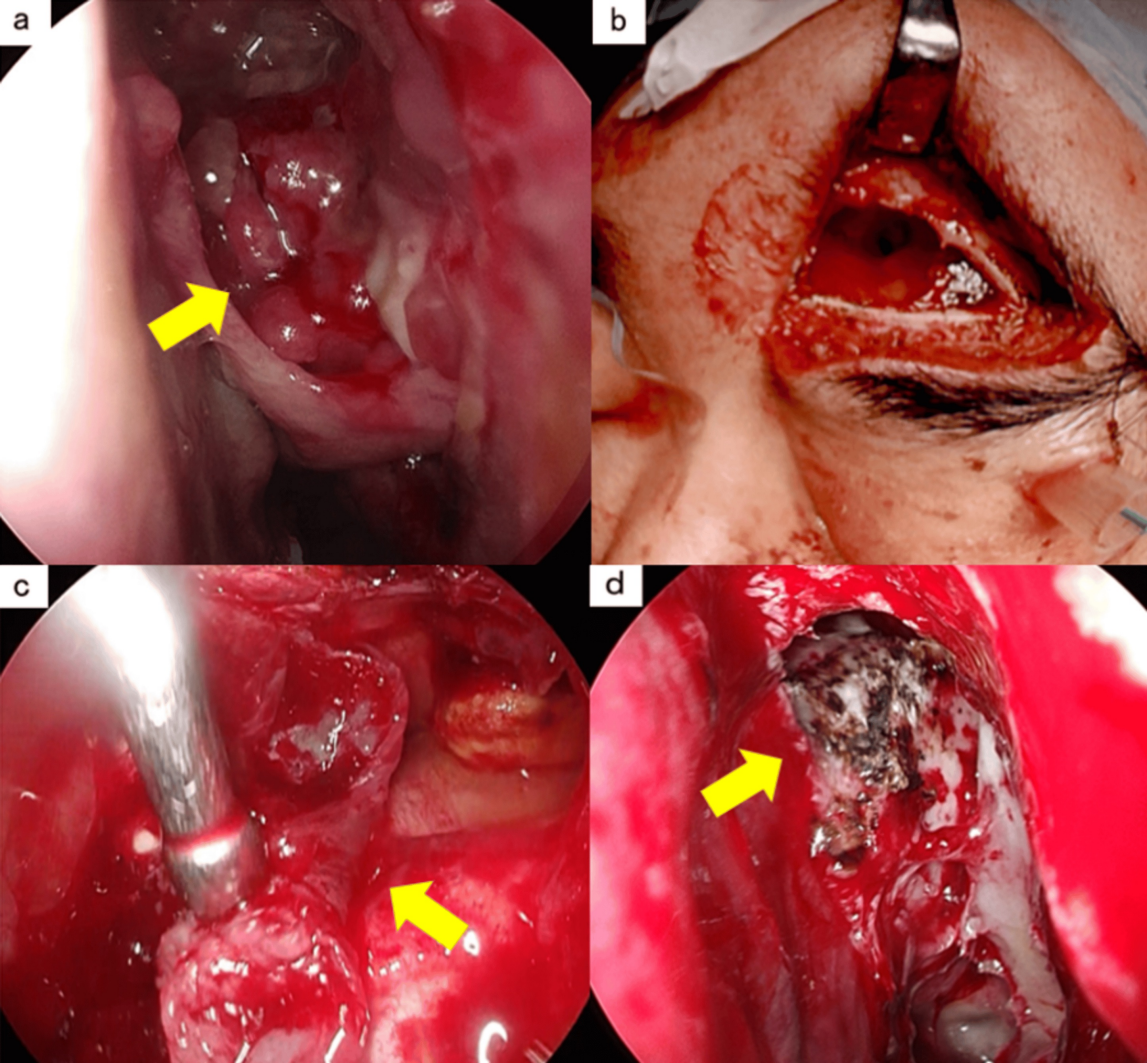
Surgical findings of ESS, Draf type IIb, and the Killian method, performed as the second stage a) The ethmoid sinus is filled with tumor. The arrow indicates the tumor. b) An incision is made in the skin just above the left eyebrow, and the frontal bone near the left frontal sinus is cut to open the frontal sinus. c) An endoscope was inserted through the frontal sinus and observed from the outside to the inside. The arrow indicates the tumor attached to the posterior wall of the frontal sinus. The tumor base attachment site is cauterized with a suction coagulator. The arrow indicates the cauterization site.

A tumor was detected in the ethmoid sinus of the left nasal cavity (Figure 5a). First, the lower part of the tumor in the ethmoid sinus was removed, and the final part of the ethmoid sinus was opened. Edema-like mucosa within the left maxillary sinus was removed as much as possible. Subsequently, the left nasal ridge was removed using a drill to obtain a view of the nasofrontal canal. The bone in the frontal sinus was removed, the nasofrontal canal was opened, the tumor base was confirmed in the nasofrontal canal, and the tumor was dissected and removed to an extent that could be confirmed under an endoscope.

Since the tumor base within the frontal sinus could not be confirmed, extranasal frontal sinus surgery was performed. A skin incision was made immediately above the left eyebrow to expose the frontal bone near the left frontal sinus, and the bone was incised to open the frontal sinus (Figure 5b). An endoscope was inserted from the frontal sinus, and the tumor was removed from the frontal sinus side of the posterior wall of the frontal sinus (Figure 5c). The attachment site at the base was cauterized using a suction coagulator (Figure 5d). The frontal bone was then returned and fixed, and the skin was sutured. After the surgery, the inside of the nose was packed with a medical sponge, and the surgery was completed.

**Figure 6:**
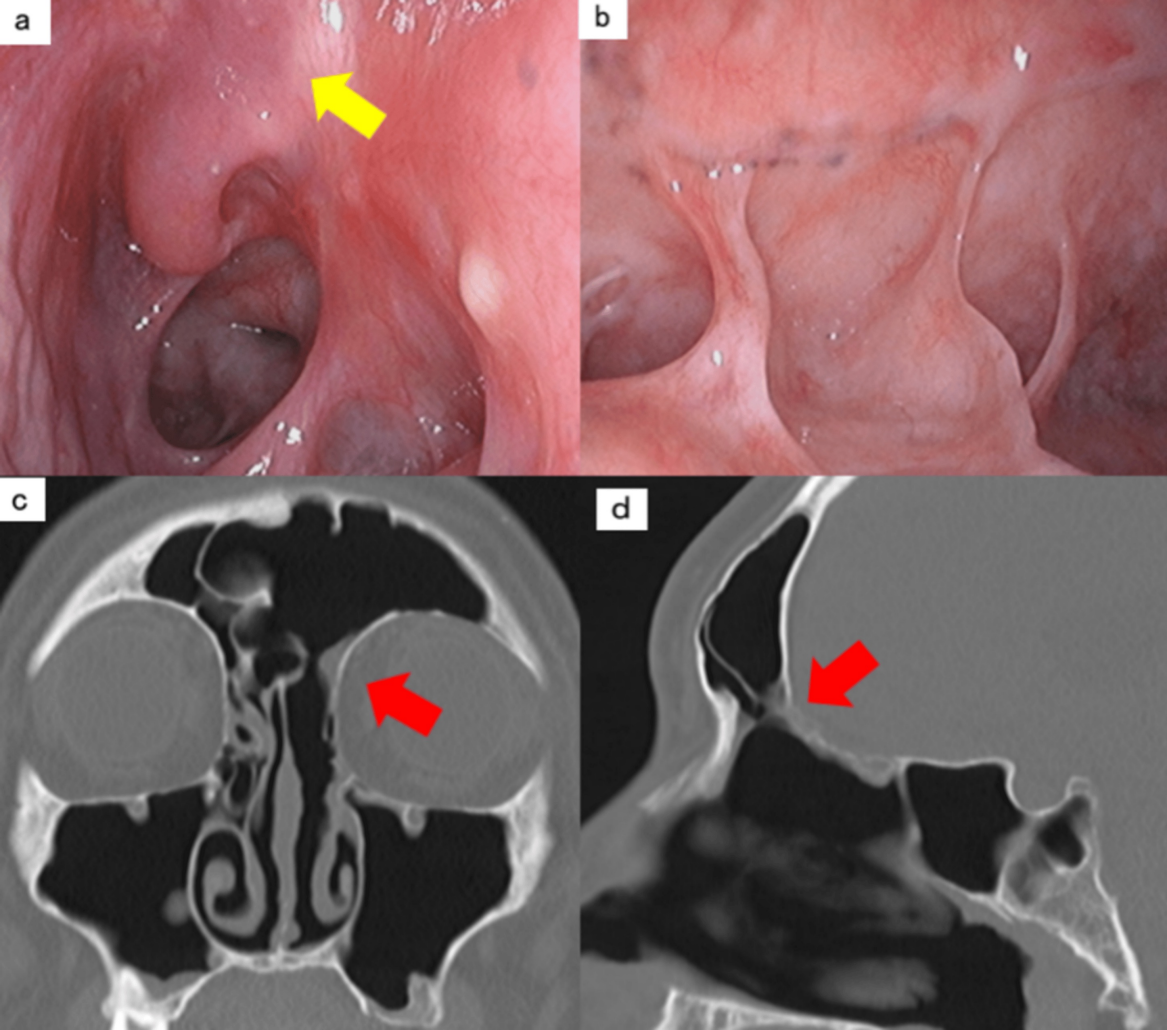
Endoscopic images and CT scans taken one year after surgery a) Endoscopic image of the ethmoid sinus. b) Endoscopic image of the frontal sinus. c) Coronal bone condition on plain CT scan of the paranasal sinuses. d) Sagittal bone condition on plain CT scan of the paranasal sinuses. All arrows indicate the area around the base of the tumor. There are no signs of recurrence in either the endoscopic or CT images.

The packing was removed on the fourth day after the second surgery. On the sixth postoperative day, the sutures on the forehead were removed, and the patient was discharged from the hospital. There was no obvious change in visual acuity after light valve treatment, and the light reflex improved after emergency surgery. After surgery, the patient's progress was confirmed by nasal endoscopy (Figures 6a, 6b) and sinus CT (Figure 6c), and no signs of recurrence were observed up to one year later.

## Discussion

Symptoms of intraorbital abscesses include swelling and redness of the eyelids, impaired eye movement, and proptosis [1,4,9]. Visual impairment does not occur as an initial symptom; however, as the intraorbital pressure increases during disease progression, further symptoms and serious disorders such as visual impairment appear. Furthermore, it is a highly urgent condition that may cause intracranial complications [10,11]. There are many reports that CT examination is useful for imaging diagnosis of intraorbital abscess, and since the abscess cavity is ring-enhanced and clearly visualized, it is easy to visualize with the use of a contrast agent, but soft tissue shadows within the orbit can also be confirmed with plain CT, so in an emergency or when it is difficult to use a contrast agent, it is also possible to make a determination with plain CT [1,9]. Regarding the treatment of nasal intraorbital complications, some cases have been cured with conservative treatment, while others require surgical treatment [12-15].

Emergency surgical treatments for nasal intraorbital complications can be broadly divided into intranasal and extranasal approaches. The method of opening the paranasal sinuses under an endonasal endoscope, removing the paper-like plate, and draining the sinuses is minimally invasive and excellent in terms of cosmetics. It can be widely used in cases located inside the orbit and within the ethmoid sinus. Extranasal incisions, in which an arc-shaped incision is made from the eyebrows to the inside of the medial canthus, can reach the medial or upper wall of the orbit and are, therefore, used when there is an abscess centered above the orbit. If the abscess has spread over a wide area, even if it can be reached using nasal endoscopy, it may not be opened sufficiently, and there is a risk of reclosure and recurrence of the abscess; therefore, a combination of intranasal and extranasal approaches is often recommended. Intraorbital abscesses often occur in childhood in boys [2,4,9,12]; however, in adults, they can be caused by inflammatory spread from the sinuses or sinus tumors, such as cysts, inverted papilloma, and malignant tumors. Secondary inflammatory spread was considered, as a differential diagnosis. Secondary sinusitis can be differentiated using imaging and pathological tests. It has been reported that MRI is useful for depicting the state of the soft tissues within the orbit in detail [1,16]. It is also useful for confirming the internal condition and tumor base and is also involved in determining treatment strategies. In addition, histopathological diagnosis is particularly important when a tumor is suspected because treatment strategies vary greatly depending on whether the tumor is benign or malignant.

In this case, the patient became aware of swelling and redness of the eyelids, and visual impairment was observed three days after symptom onset. Plain CT and MRI led to the diagnosis of a nasal sinus tumor/abscess, intraorbital abscess, and intracranial epidural abscess. The abscess had extended into the orbit, and the patient's symptoms worsened after conservative treatment with antibiotics. Therefore, surgery was indicated, and the patient's vision was impaired; therefore, emergency drainage was deemed necessary.

Because the abscess inside, above, and outside the orbit affected visual acuity and because the nasal sinuses were filled with the tumor, it was difficult to approach the orbit from within the nose; therefore, an extra nasal incision was performed to improve visual function. MRI revealed a suspected tumor lesion, so an intranasal tumor biopsy was performed to diagnose the tumor, and intranasal drainage was performed. In addition to spreading the abscess over a wide area, the presence of a tumor may cause the abscess to divide into the nasal cavity or frontal sinus. Therefore, we believe that a combination of external approaches would be effective.

The advantage of performing surgery in two stages is that, by making a definitive diagnosis, the treatment policy for the tumor can be clarified. Although inversion papilloma is a benign tumor, approximately 1.9-27% of cases develop into cancer [17]. The risk of recurrence and malignant progression can be reduced by confirming the base and removing the tumor. Malignant tumors may require tumor removal with margins for complete removal or treatment with chemotherapy or radiotherapy. In this case, a malignant mass was suspected, and emergency surgery was performed in two stages with the aim of preserving visual function through drainage and establishing a diagnosis. While controlling the abscess at an early stage by emergency surgery, it is possible to make a definitive diagnosis of entropic papilloma, check the bottom using MRI, and treat it postoperatively with an external nasal incision. Complete tumor removal was possible. Even after the second surgery, the patient was diagnosed with inversion papilloma. Postoperative follow-up was continued, and no recurrence has been observed to date. For intraorbital abscesses caused by nasal/sinus tumors, a two-stage treatment is considered an option, first focusing on improving visual function, drainage, and definitive diagnosis and then moving on to radical treatment.

Some limitations should be noted. Our findings are based on a single case and may not be generalizable to other patients or populations. Depending on the tumor stage at diagnosis, our treatment method may not be effective in some cases. Individual patient comorbidities should also be taken into consideration.

## Conclusions

Intraorbital abscess is a highly urgent disease that can cause visual impairment and intracranial complications. A patient experienced an intraorbital abscess caused by a frontal sinus varus inverted papilloma. Two-stage treatment was effective: first, emergency drainage using extranasal/intranasal approaches was performed to improve visual function and for diagnosis, and after a definitive diagnosis, radical surgery was performed.
